# COX-2/PGE_2_ axis blockade with celecoxib enhances anti-PD-1 efficacy by activating natural killer cells for residual hepatocellular carcinoma after radiofrequency ablation

**DOI:** 10.1186/s13046-025-03582-6

**Published:** 2025-12-17

**Authors:** Yu Lei, Yaowei Bai, Xiatong Bai, Bo Sun, Licheng Zhu, Wenlong Wu, Yang Su, Hongsen Zhang, Yingliang Wang, Chuansheng Zheng

**Affiliations:** 1 Department of Radiology, Union Hospital, Tongji Medical College, Huazhong University of Science and Technology, Jiefang Avenue #1277, Wuhan, 430022 China; 2 Hubei Provincial Clinical Research Center for Precision Radiology & Interventional Medicine, Wuhan, 430022 China; 3 Hubei Province Key Laboratory of Molecular Imaging, Wuhan, 430022 China

**Keywords:** Celecoxib, αPD-1, NK cells, Incomplete radiofrequency ablation, Hepatocellular carcinoma

## Abstract

**Background and aims:**

Radiofrequency ablation (RFA) is an effective treatment for hepatocellular carcinoma (HCC), but incomplete ablation and recurrence of residual tumors remain significant challenges, partly due to local inflammation and elevated COX-2 levels in the tumor microenvironment. This study aims to investigate the potential of combining celecoxib, a COX-2 inhibitor, with anti-PD-1 monoclonal antibody (αPD-1) to enhance anti-tumor efficacy and activate immune responses.

**Methods:**

In vitro, ELISA was used to assess the effects of radiofrequency heat treatment and celecoxib on PGE_2_ secretion by Hepa1-6 cells. Scanning electron microscopy, flow cytometry, and CCK-8 assays were employed to evaluate the function and cytotoxic activity of NK92 cells against Hepa1-6 cells. In vivo, orthotopic HCC mice were divided into five groups to evaluate tumor volume, pathology, and survival. The role of celecoxib in the COX-2/PGE_2_/NK cell axis and its impact on NK cell immune function were investigated.

**Results:**

In vitro experiments showed that celecoxib reversed PGE_2_-mediated suppression of NK cell function and cytotoxic activity against HCC cells by inhibiting PGE_2_ secretion. In vivo, orthotopic HCC mice treated with celecoxib and αPD-1 exhibited significantly reduced tumor volumes, attenuated the infiltration and activation of NK cells, and prolonged survival compared to control groups. The combination therapy also demonstrated a notable abscopal-like effect, inhibiting metastatic tumor growth and activating systemic immunity.

**Conclusions:**

Taken together, these findings suggest that celecoxib combined with αPD-1 represents a promising strategy for treating residual HCC after RFA, with enhanced anti-tumor effects and good safety.

**Graphical abstract:**

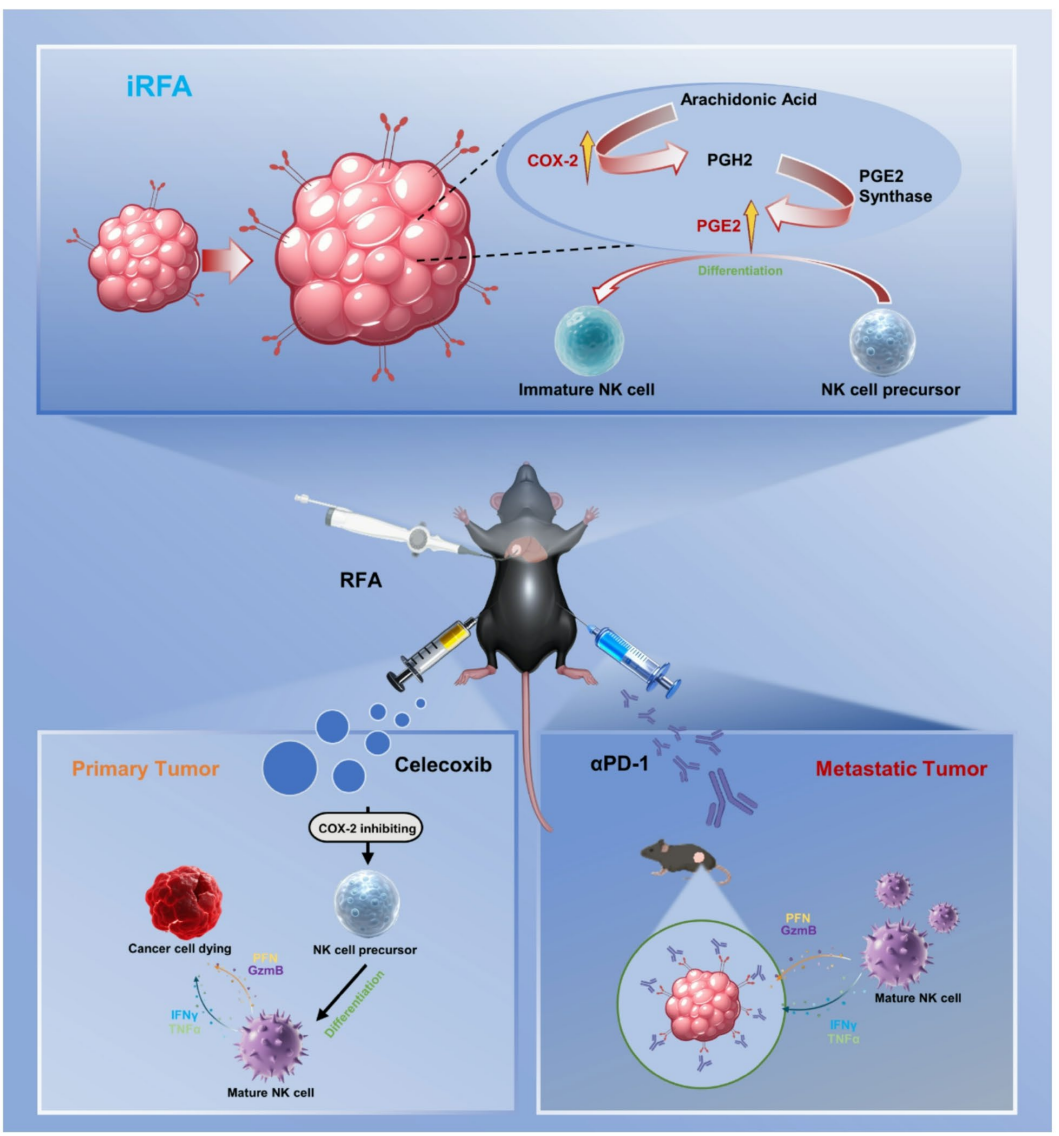

**Supplementary Information:**

The online version contains supplementary material available at 10.1186/s13046-025-03582-6.

## Introduction

Liver cancer ranks among the most prevalent and lethal forms of cancer globally [1]. In 2022, there were 865,269 new cases and 757,948 individuals died due to liver cancer [2]. Among the various types of liver cancer, hepatocellular carcinoma (HCC) stands out as the most frequently occurring form [3].

Radiofrequency ablation (RFA) is widely used as a curative treatment for early-stage HCC classified as BCLC-A[4]. However, it remains a formidable challenge to achieve complete ablation of HCCs larger than 3 cm in diameter or with irregular morphology. Due to the heat-sink effect, the temperature around the ablation center is relatively low, which can lead to the survival of viable tumor tissue. This is termed as incomplete radiofrequency ablation (iRFA) [5, 6]. Worse still, iRFA can accelerate tumor progression and even promote metastasis [7]. Studies have shown that iRFA causes an increase in Cyclooxygenase-2 (COX-2) levels, which leads to increased secretion of prostaglandin E_2_ (PGE_2_) via the COX-2/PGE_2_ axis [8]. PGE₂ recruits neutrophils that suppress antitumor immunity [9]. Additionally, tumor-derived PGE_2_ impairs production of antitumor cytokines, and downregulates chemokine receptor expression in cDC1 cells, rendering them unable to effectively present antigen and initiate cytotoxic responses [10]. Natural killer (NK) cells, innate lymphocytes that inherently recognize and kill infected or malignant cells, have recently emerged as promising targets for cancer immunotherapy [11]. Chloe Patterson et al. demonstrated that PGE₂ reduces activating-receptor expression on NK cells, delays polarization, and diminishes the kinetic interaction between NK cells and cancer cells [11]. Specifically inhibiting the elevated COX-2 activity after iRFA to reduce PGE_2_ production could potentially resolve these challenges.

Programmed Cell Death Protein 1 (PD-1) on immune cells and its ligand PD-L1 on tumor cells function as a critical immune “brake” that blocks immune-cell activation, proliferation, and cytokine production [12, 13]. In recent years, immunotherapies targeting the PD-1/PD-L1 axis have achieved unprecedented success across multiple human cancers [14]. For intermediate and advanced HCC, guidelines recommend the use of immune checkpoint inhibitor (ICI) therapy [15]. Several studies have reported that iRFA upregulates PD-L1 expression, thereby enhancing tumor immune evasion and accelerating progression [16, 17]. Therefore, anti-PD-1 checkpoint blockade represents a rational treatment strategy.

In summary, our study employed the selective COX-2 inhibitor celecoxib to suppress COX-2 activity, reduce PGE₂ secretion, and thereby preserve the antitumor activity of immune cells—especially NK cells. Combined with a PD-1 monoclonal antibody (αPD-1) to block PD-1/PD-L1-mediated tumor immune escape, we investigated the efficacy against residual tumors after local iRFA and the impact on systemic antitumor immunity.

## Methods and materials

### Patients and sample collection

We enrolled ten consecutive patients with BCLC stage-A HCC who were managed at the Department of Interventional Radiology, Union Hospital, Tongji Medical College, Huazhong University of Science and Technology. Five patients were treat-naïve, and five patients had undergone a single prior RFA treatment. Tumor tissue was obtained by surgical resection and histologically verified as HCC. Written informed consent was provided by every participant.

### Cell culture and drug treatments

Hepa1–6 cells (Chinese Academy of Sciences, Shanghai, China) were cultured in DMEM medium (Procell Life Science & Technology Co., Ltd., Hubei, China) supplemented with 10% fetal bovine serum (FBS) (Gibco, NY, USA). NK-92 cells (Procell Life Science & Technology Co., Ltd., Hubei, China) were cultured in α-MEM containing 12.5% FBS, 12.5% horse serum, 0.2 mM inositol, 0.02 mM folic acid, 0.1 mM β-mercaptoethanol, 1% Penicillin-Streptomycin solution and 100U/mL IL-2. All cells were maintained at 37 ℃ in a humidified incubator containing 5% carbon dioxide. For the pertinent investigations, NK92 cell line was treated with PGE_2_ (Aladdin Biochem Technology Co., Ltd., Shanghai, China) when they had reached 80–90% confluence.

### Detection of PGE_2_ levels by enzyme-linked immunosorbent assay (ELISA)

Hepa1-6 cells were seeded at a density of 20,000 cells per well in a 6-well plate and assigned to six groups, each with three replicates. The groups were as follows: (1) phosphate-buffered saline (PBS) control; (2) low-dose celecoxib (20 µM) (Aladdin Biochem Technology Co., Ltd., Shanghai, China); (3) high-dose celecoxib (40 µM); (4) radiofrequency hyperthermia (RFH) treatment alone; (5) RFH combined with low-dose celecoxib (20 µM); (6) RFH combined with high-dose celecoxib (40 µM). Celecoxib was added to the wells containing Hepa1-6 cells, followed immediately by RFH treatment. The RFH procedure involved placing the 6-well plate horizontally in a water bath and maintaining a temperature of 46 °C for 10 min. The concentration of PGE_2_ in the supernatants of each group was measured using a PGE_2_ ELISA kit (eBiosciences).

Human HCC tissues were first homogenized, and the supernatants were collected to determine PGE₂ levels according to the manufacturer’s instructions of a PGE₂ ELISA kit (eBiosciences).

### Scanning electron microscopy (SEM)

NK92 cells were seeded at a density of 10,000 cells/mL and allocated into the following three groups: (1) treated with PBS for 48 h; (2) treated with low-dose PGE_2_ (20 µM) for 48 h; and (3) treated with high-dose PGE_2_ (40 µM) for 48 h. The morphology of the treated NK92 cells was examined using SEM (Hitachi Regulus 8100).

### Cytotoxicity assay of PGE_2_-treated NK92 cells

Hepa1-6 cells were seeded in 96-well plates at a density of 5,000 cells per milliliter and incubated for 12 h to enable adherence. Following this, NK92 cells that had been treated with varying concentrations of PGE_2_ were co-cultured with Hepa1-6 cells at an effector-to-target ratio of 5:1. The co-culture groups were as follows: (1) Control group: Hepa1-6 cells co-cultured with NK92 cells treated with PBS; (2) Hepa1-6 cells co-cultured with NK92 cells treated with low-dose PGE_2_; (3) Hepa1-6 cells co-cultured with NK92 cells treated with high-dose PGE_2_. After 48 h of co-culture, the supernatant was collected and centrifuged. The resulting supernatant was analyzed using ELISA kits to measure the concentration of TNF-α and IFN-γ (eBiosciences). The precipitated NK92 cells were collected and analyzed by flow cytometry for the expression of activating receptors (NKp30, NKp46, NKG2D) and inhibitory receptors (NKB1). Hepa1-6 cell viability was assessed using a CCK-8 kit (Elabscience, China). Adherent cells in the wells were digested with 0.25% trypsin, and the digestion was terminated by adding culture medium. The cells were then washed twice with PBS, collected by centrifugation, and resuspended in 100µL of binding buffer. A mixture containing 2.5µL of Annexin V-APC and 2.5µL of DAPI (Annexin V-APC/DAPI Apoptosis Detection Kit, Elabscience, China) was added, and each group of cells was incubated in the dark for 15 min. Apoptosis rates were determined by flow cytometry (Sony ID7000, Inc). Additionally, the proliferation and migration activities of the remaining viable Hepa1-6 cells were evaluated using colony formation and scratch assays.

### Colony formation assay and scratch wound healing assay

The colony formation assay was used to evaluate cell survival and proliferative capacity, while the scratch wound healing assay was employed to assess cell migration ability. Detailed experimental procedures are provided in the supplementary Appendix 1.

### Tumor mouse models and treatment

Five-week-old healthy male C57BL/6 mice (BIONT Biological Technology Co., Ltd., Hubei, China) were used in our study. All animal experiments were conducted with the approval of the Animal Ethics Committee of our institute and in strict accordance with the ARRIVE guidelines. After anesthesia with an intraperitoneal injection of sodium pentobarbital at 45 mg/kg, Hepa1-6 cells (1 × 10⁶) were injected into the livers of the mice. After two weeks, when the tumor volume reached approximately 200 mm³, the tumor model was deemed successfully established.

Subsequently, the mice were randomly assigned to five groups (*n* = 5 per group, totaling 25 mice) and received the following treatments: (1) Sham treatment of the tumor with an intraperitoneal injection of 100µL PBS (control group); (2) iRFA treatment at 70 °C for 2 min, followed by an intraperitoneal injection of 100 µL PBS; (3) iRFA treatment followed by an intraperitoneal injection of 100µL αPD-1 (clone RMP1-14, BioXCell); (4) iRFA treatment followed by an intraperitoneal injection of 100µL celecoxib; (5) iRFA treatment followed by intraperitoneal injections of both 100µL αPD-1 and 100µL celecoxib. The αPD-1 was administered repeatedly on days 1, 4, 7, 10, and 13 after the initial treatment. Celecoxib and PBS were administered intraperitoneally once daily. For the iRFA treatment, mice were anesthetized with 1–3% isoflurane (RWD Life Science Co., Ltd, Shenzhen, China) via inhalation. To perform the procedure, an incision was made to access the abdominal cavity, and a 17-gauge monopolar ablation electrode (RITA Medical Systems, Inc., Mountain View, CA, USA) was inserted into the tumor through its dorsal aspect. The ablation was carried out at a temperature setting of 70 °C for a duration of 2 min. Our preliminary study demonstrated that this specific ablation protocol established an incomplete tumor ablation model, characterized by approximately 70% tumor necrosis and 30% residual viable tumor tissue [18]. On day 21, tumor-bearing mice were euthanized, and tumor volume was calculated using the formula: volume (mm³) = width² × length/2. Tumor samples were harvested and weighed for further analysis.

### Haematoxylin–eosin (HE), immunohistochemistry and immunofluorescence staining

Tissue samples obtained from patients and the mouse model were processed for histological analysis. They were fixed in paraffin and sectioned at a thickness of 4 mm, following the standard protocols for HE staining and immunohistochemistry. For apoptosis detection, the sections were subjected to terminal deoxynucleotidyl transferase-mediated dUTP-biotin nick end labeling (TUNEL) staining using the 3’-diaminobenzidine (DAB) (SA-HRP) TUNEL Apoptosis Detection Kit (Servicebio, China). The sections were then incubated with primary antibodies against Ki-67 (ab16667, 1:200 dilution; Abcam, Cambridge, UK), PD-L1 (ab213524, 1:250 dilution; Abcam, Cambridge, UK), and COX-2 antibodies (610204, 1:200 dilution, BD Biosciences Pharmingen, San Diego, CA, USA) in 0.01 mol/L citrate buffer at 4 °C overnight. On the following day, immunodetection was performed using DAB as per the manufacturer’s instructions.

Immunofluorescence staining was performed on patient tissue sections to visualize NK cells. Sections were deparaffinized, subjected to antigen retrieval and blocked for non-specific binding according to the manufacturer’s instructions. The tissue slices were then incubated with an CD56 primary antibody, followed by a fluorescent secondary antibody. Fluorescence images were captured using a fluorescence microscope.

### Western blot

Total protein was extracted from cells using a mammalian total protein extraction kit and protease inhibitor cocktail (Transgen, Beijing, China). Briefly, proteins were separated by 10% SDS-polyacrylamide gel electrophoresis (SDS-PAGE) and transferred to PVDF membranes. Specific primary antibodies were used to detect TRAIL (GB11413-100, 1:1000 dilution, Servicebio, China), FASL (GB11090-100, 1:1000 dilution, Servicebio, China), and COX-2 (GB115672-100, 1:1000 dilution, Servicebio, China). The PVDF membranes were incubated with these primary antibodies at 4 °C overnight. Subsequently, the membranes were treated with horseradish peroxidase-conjugated secondary antibodies at room temperature for 2 h. Finally, Western blot bands were visualized using an enhanced chemiluminescence (ECL) detection system (Thermo Fisher, USA).

### Quantitative reverse transcriptase-polymerase chain reaction (qRT-PCR)

Total RNA was extracted from the samples using the Total RNA Extraction Kit (Solarbo, China). Following this, the extracted RNA was reverse-transcribed into cDNA using the first-strand complementary DNA synthesis kit (Invitrogen, USA) as per the manufacturer’s instructions. The expression levels of cytolytic effector molecules, including TRAIL, FASL, perforin, and granzyme B, were quantitatively analyzed in tumor tissues using the Premix Ex Taq SYBR Green polymerase chain reaction kit (Sigma-Aldrich, Germany). GAPDH was used as an internal normalization control. Relative gene expression was initially analyzed using the ΔCT method, which calculates the ratio of target gene expression to that of GAPDH. Subsequently, the relative expression levels were normalized to the baseline expression level of the control group. Details of all primers used for qRT-PCR can be found in Supplementary Table S1.

### ELISA detection of cytokines

Tumor tissues and spleens were homogenized, and the tissue supernatants were collected following low-temperature ultracentrifugation for subsequent analysis. Blood was collected from the ophthalmic artery into tubes containing ethylenediaminetetraacetic acid (EDTA). Plasma was separated by centrifugation at 1000 g for 10 min and stored for further analysis. The concentrations of PGE_2_, IL-2, TNF-α, and IFN-γ in tumor tissues, spleens, and plasma were quantified using high-sensitivity ELISA kits (eBiosciences).

### Flow cytometrical analysis

For flow cytometric analysis of tumor and spleen tissues, tumor tissues and spleens were harvested and homogenized. Tumor tissues were digested with a collagenase and hyaluronidase solution, mechanically dissociated into single-cell suspensions, and filtered through cell strainers. The suspensions were then resuspended in Hank’s medium containing 1% FBS for further analysis. Spleen cells were processed by red blood cell lysis, washed, and fixed. Antibodies used included CD45 (557659), CD3 (564008), NK1.1 (552878), CD11b (557396), and PD-1 (551892) from BD Biosciences Pharmingen (San Diego, CA, USA), as well as CD122 (123214) and CD27 (124241) from BioLegend (San Diego, CA, USA). Flow cytometric analysis was performed using a FACS flow cytometer (Canto II, BD), and data were analyzed using FlowJo software.

### Abscopal-like effect assessment and rechallenge test

To investigate the abscopal-like effect, Hepa1-6 cells (1 × 10⁶) were subcutaneously injected into the right dorsal flank of mice following anesthesia induced by an intraperitoneal injection of sodium pentobarbital at 45 mg/kg. Throughout the treatment period, these subcutaneous tumors were not subjected to local treatment. For the rechallenge test, mice that survived the entire observation period in the RFA and combination therapy groups were re-injected with Hepa1-6 cells (1 × 10⁶) subcutaneously into the right dorsal flank following anesthesia. The rechallenge test was performed to determine whether combination therapy enhanced antitumor immunity against HCC tumors. The re-implanted tumors were measured weekly for 3 weeks and were harvested, measured, and weighed at the end of the 3-week period.

### Safety assessment

Fourteen days post-treatment, biochemical analyses were conducted to assess alanine aminotransferase (ALT), aspartate aminotransferase (AST), creatine kinase MB (CKMB), and creatinine (CREA). Additionally, normal tissues from the heart, liver, spleen, lung, and kidney were stained with HE to evaluate the safety of the treatments.

### Statistical analysis

All statistical analyses were conducted using SPSS 24.0 software (IBM, Chicago, IL). Scientific graphs were created by GraphPad Prism 9.5.0 (San Diego, CA, USA). Data are presented as mean ± standard deviation (SD). An unpaired two-tailed Student’s t-test was performed for two-group comparisons, and analysis of variance (ANOVA) followed by Tukey’s multiple comparisons test was performed for multiple group comparisons. Survival data were estimated using the Kaplan-Meier method and analyzed by the log-rank test. A p-value of less than 0.05 was considered statistically significant.

## Results

### COX-2/PGE2 axis activation and NK cell suppression in human HCC after RFA

**Fig. 1:**
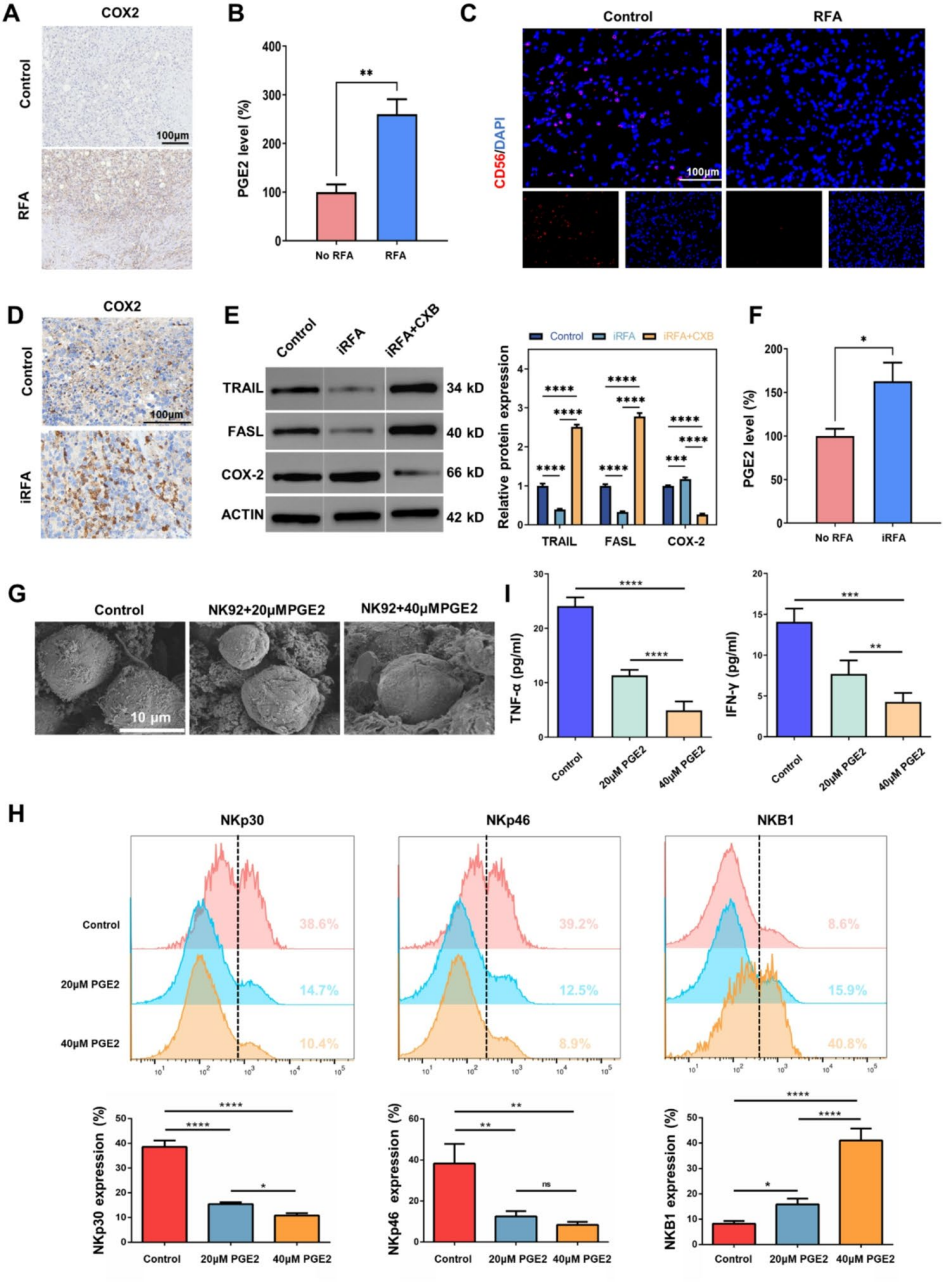
Changes in the COX-2/PGE_2_/NK cell axis after iRFA. **A** Immunohistochemical staining of COX-2 expression in human HCC with or without RFA. **B** Intratumoral PGE_2_ levels in human HCC patients with or without prior RFA therapy. **C** Immunofluorescence micrographs of NK cell infiltration in human HCC. **D** Immunohistochemical staining of COX-2 expression in murine orthotopic HCC models with or without iRFA. **E** Western blot analysis of TRAIL, FASL and COX-2 expression in murine orthotopic HCC tumor tissues from different groups. (F) ELISA analysis of PGE_2_ secretion by NK92 cells treated with or without iRFA treatment. **G** SEM images of NK92 cells treated with different concentrations of PGE_2_. **H** Flow cytometry analysis and corresponding bar charts of activating and inhibitory receptors on NK92 cells treated with PGE_2_. **I** ELISA analysis of IFN-γ and TNF-α secretion by NK92 cells treated with PGE_2_. (*p < 0.05; **p < 0.01; ***p < 0.001; ****p < 0.0001; RFA, radiofrequency ablation; iRFA, incomplete radiofrequency ablation; PGE_2_, prostaglandin E2)

To determine whether RFA alters the COX-2/PGE2 axis in patients, we compared human HCC tumor samples that had undergone RFA with untreated human HCC specimens. Immunohistochemistry revealed markedly higher COX-2 expression in post-RFA sections than in controls (Fig. 1A). ELISA further revealed a 2.5-fold surge in intratumoral PGE2 levels following RFA (Fig. 1B). Meanwhile, we employed immunofluorescence imaging to scrutinize the extent of NK cell infiltration within residual HCC tumor tissues. The obtained images revealed that NK cell infiltration was markedly diminished in the tumor tissues following RFA (Fig. 1C). Collectively, these human data demonstrate that RFA simultaneously up-regulates the COX-2/PGE2 pathway and inhibited NK cell infiltration within residual HCC.

### In vitro and in vivo exploration of the potential mechanisms

Subsequently, we performed investigations in an orthotopic mouse model and in cultured murine Hepa1-6 cells to explore the mechanisms underlying post-RFA alterations. In an orthotopic HCC mouse model, we simulated iRFA treatment for liver tumor. IHC and Western blot were employed to assess COX-2 expression levels in tumor tissues following iRFA. Both methodologies demonstrated a pronounced up-regulation of COX-2 after iRFA (Fig. 1D and E). To investigate the effect of RFA on PGE_2_ secretion, Hepa1-6 cells were thermally treated in vitro using a water bath. ELISA results showed that thermal treatment promoted PGE_2_ secretion by Hepa1-6 cells (Fig. 1F).

To investigate the impact of elevated PGE_2_ after iRFA on NK cells, NK92 cells were incubated with different concentrations of PGE_2_ in vitro. Following a 48-hour incubation period, SEM imaging revealed notable morphological changes in PGE_2_-treated NK92 cells compared to the PBS control group (Fig. 1G). These changes were characterized by decreased cell clustering and the disappearance of typical synapse-like structures. Flow cytometry was employed to evaluate the expression levels of activating and inhibitory receptors on the surface of NK92 cells. PGE2-treated NK92 cells showed significantly lower levels of the activating receptors NKp30 and NKp46. Concurrently, expression of the inhibitory receptor NKB1 was markedly increased relative to the control group (Fig. 1H). Taken together, these findings demonstrate that elevated PGE₂ after iRFA suppresses NK-cell activity and function.

**Fig. 2:**
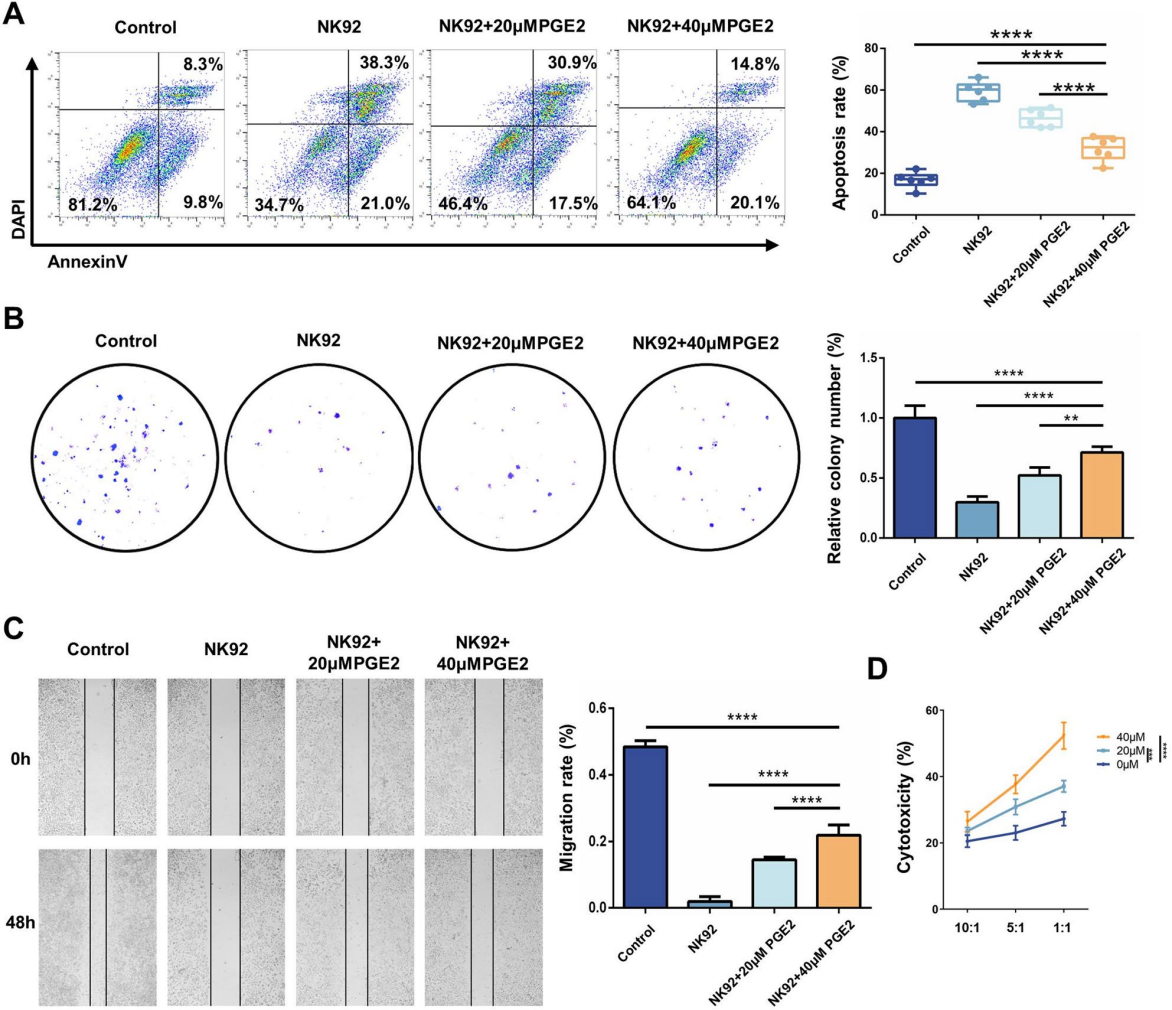
PGE_2_ suppresses the antitumor activity of NK cells. **A** Flow cytometry analysis of apoptosis in Hepa1-6 cells co-cultured with NK92 cells treated with different concentrations of PGE_2_. **B** Colony formation assay evaluating the proliferation of Hepa1-6 cells co-cultured with NK92 cells treated with different concentrations of PGE_2_. **C** Wound healing assay evaluating the migration of Hepa1-6 cells co-cultured with NK92 cells treated with different concentrations of PGE_2_. **D** Cytotoxicity of NK92 cells treated with different concentrations of PGE_2_ (0, 20, and 40 µM) against Hepa1-6 cells at indicated effector-to-target (E/T) ratios. (*n* = 3, *p < 0.05; **p < 0.01; ***p < 0.001; ****p < 0.0001; PGE_2_, prostaglandin E2; TNF-α, tumor necrosis factor-α; IFN-γ, interferon-γ)

To investigate the effector functions of NK92 cells after PGE_2_ treatment, NK92 cells from three different treatment groups were employed as effector cells and co-cultured with Hepa1-6 cells (target cells) for 4 h. ELISA was used to measure the levels of effector molecules and downstream chemokines secreted by NK cells in the co-culture supernatant. The results demonstrated that PGE_2_-treated NK92 cells exhibited significantly lower secretion of IFN-γ and TNF-α compared to the control group (Fig. 1I). As illustrated in Fig. 2A, flow cytometry revealed that apoptosis levels in Hepa1-6 cells co-cultured with NK92 cells were significantly elevated compared to the control group. However, co-culture with PGE_2_-treated NK92 cells resulted in significantly reduced apoptosis of Hepa1-6 cells compared to co-culture with untreated NK92 cells. Colony formation and scratch assays indicated that co-culture with NK92 cells inhibited the migration of Hepa1-6 cells. In contrast, Hepa1-6 cells co-cultured with PGE_2_-treated NK92 cells exhibited enhanced proliferation and migration capabilities (Fig. 2B and C). These findings suggest that PGE_2_ can impair the antitumor activity of NK92 cells. We further analyzed the cytotoxic activity of NK92 cells against tumor cells under different effector-to-target (E/T) ratios (1:1, 5:1, 10:1). As illustrated in Fig. 2D, PGE_2_-treated NK92 cells exhibited significantly diminished cytotoxic activity across all tested E/T ratios, with the inhibitory effect being dose-dependent.

### Celecoxib reverses iRFA-induced alterations

**Fig. 3:**
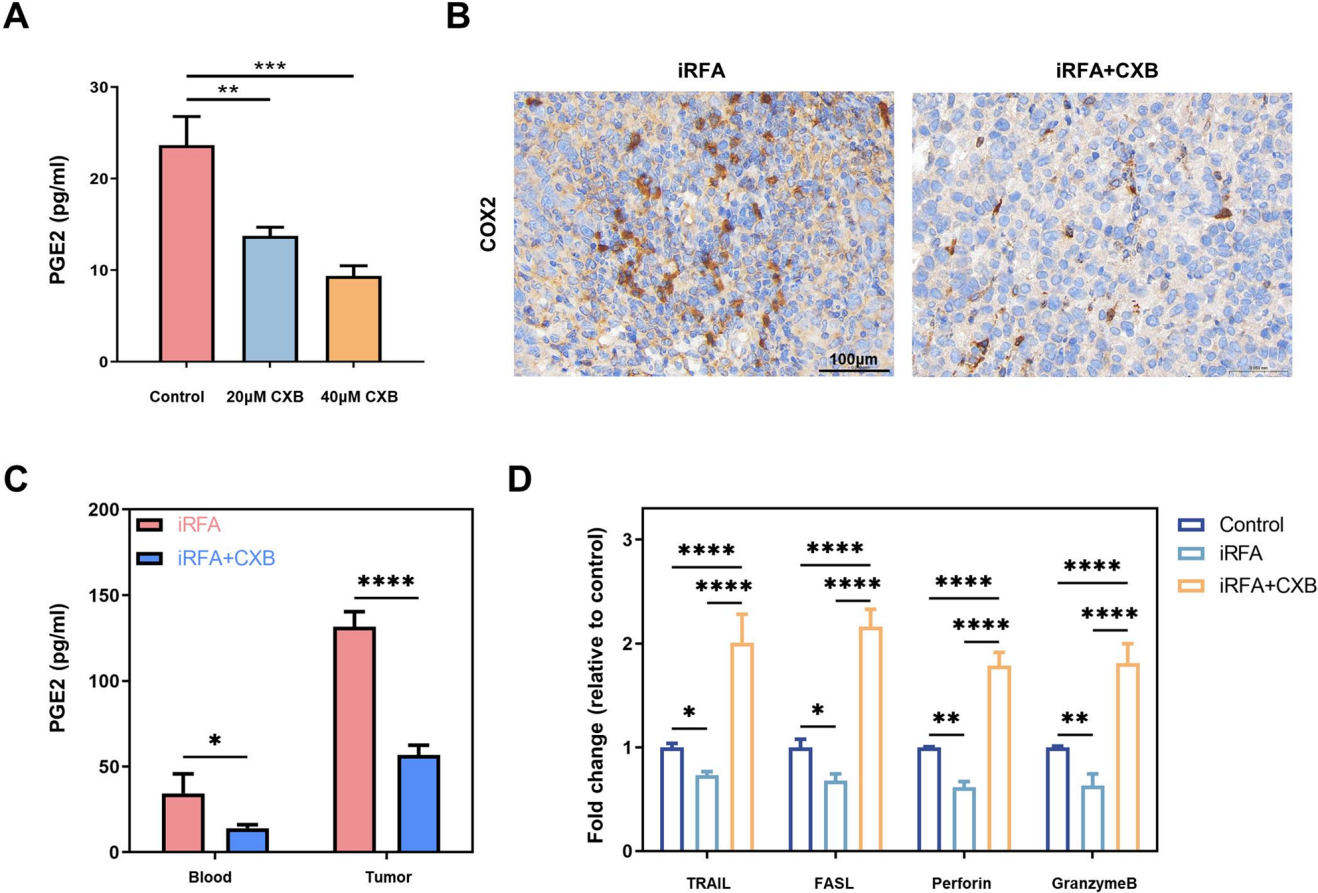
Celecoxib reverses post-iRFA alterations. **A** ELISA analysis of PGE_2_ secretion by NK92 cells after ablation and treated with different concentrations of celecoxib. **B** Immunohistochemical staining for COX-2 expression in tumors from iRFA-treated mice ± celecoxib. **C** PGE_2_ concentrations in peripheral blood and tumor tissues of iRFA-treated mice ± celecoxib. **D** qRT-PCR analysis of mRNA expression levels of cytolytic effector molecules (TRAIL, FASL, Perforin, Granzyme B) in tumor tissues. (*n* = 5, *p < 0.05; **p < 0.01; ***p < 0.001; ****p < 0.0001; iRFA, incomplete radiofrequency ablation; αPD-1, anti-PD-1 antibody; CXB, celecoxib)

Hepa1-6 cells subjected to thermal stress were treated with escalating doses of celecoxib. ELISA revealed a dose-dependent reduction in supernatant PGE₂ concentration (Fig. 3A). In the orthotopic HCC mouse model, animals received iRFA followed by daily intraperitoneal celecoxib. Tumoral COX-2 expression was markedly lower in celecoxib-treated mice than in iRFA group (Fig. 3B). Consistently, both serum and intratumoral PGE₂ levels were significantly decreased by celecoxib administration (Fig. 3C). To assess NK cell effector function, cytolytic molecule expression was quantified by qRT-PCR and Western blot. Transcript levels of TRAIL, FASL, perforin and granzyme B were lowest in the iRFA group and were restored to levels exceeding those of untreated controls after celecoxib therapy (Fig. 3D). Western blot corroborated these findings at the protein level for TRAIL and FASL (Fig. 3E).

### Efficacy evaluation of celecoxib combined with αPD-1 for residual tumors after iRFA of HCC

**Fig. 4:**
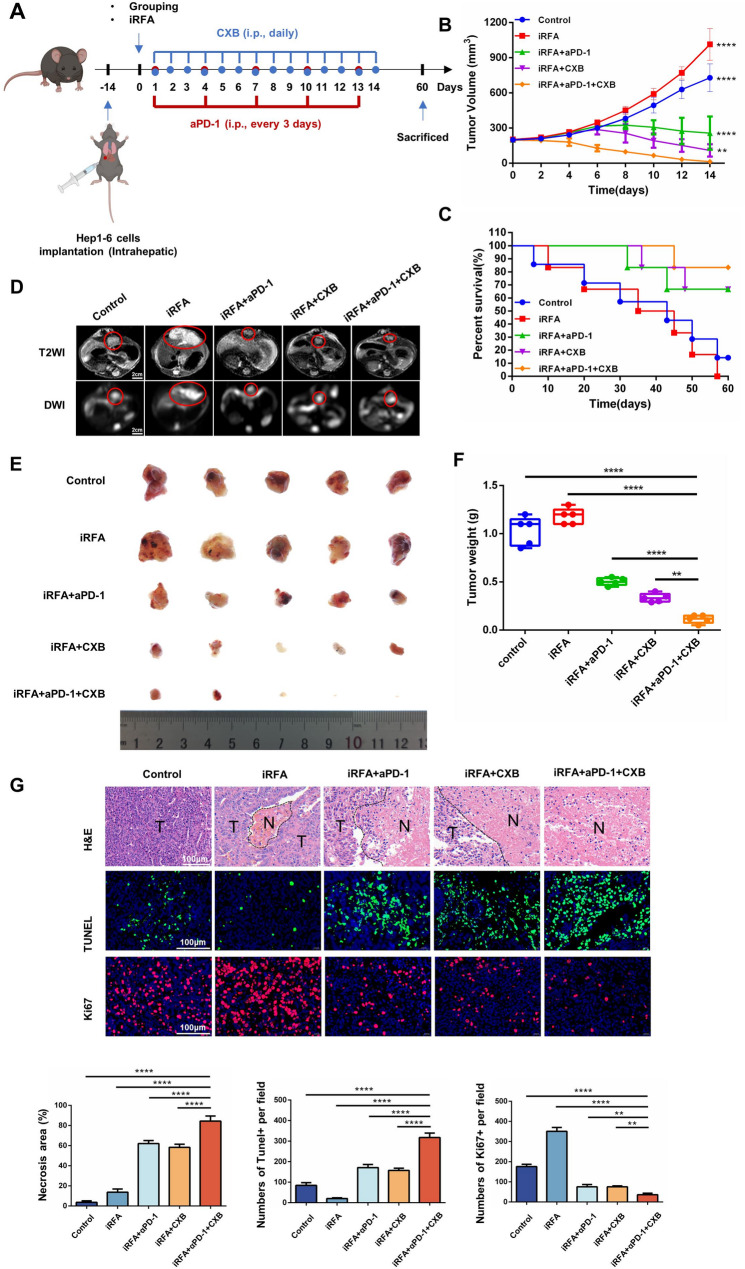
Efficacy of celecoxib and αPD-1 combination therapy in a mouse orthotopic HCC model. **A** Schematic diagram of the experimental design for in vivo efficacy evaluation of celecoxib and αPD-1 combination therapy in a mouse orthotopic HCC model. **B** Quantitative analysis of tumor volumes in different treatment groups. Data are presented as mean ± SD. **C** Kaplan–Meier survival curves showing the lifespan of tumor-bearing mice in different groups. **D** Magnetic resonance imaging (MRI) showing tumor volumes in different treatment groups 14 days after treatment. **E** Images of harvested tumors from different treatment groups. **F** Tumor weights in different treatment groups. **G** HE, TUNEL and Ki-67 staining analysis of tumors. (*n* = 5, *p < 0.05; **p < 0.01; ***p < 0.001; ****p < 0.0001; iRFA, incomplete radiofrequency ablation; αPD-1, anti-PD-1 antibody; CXB, celecoxib)

As shown in Fig. 4A, an experiment was designed to validate the in vivo efficacy of celecoxib and αPD-1 combination therapy in a mouse model of liver cancer. These mice were randomly allocated into five groups. In the mouse liver lobes, HCC was detected in situ after 14 days. The average tumor volumes in the five groups were 192.56 ± 3.31 mm³, 198.12 ± 1.55 mm³, 201.87 ± 3.12 mm³, 194.67 ± 3.89 mm³, and 202.34 ± 2.13 mm³, respectively, with no significant differences among them (*p* = 0.999). Tumor growth was monitored in each of the five groups after the respective treatments. As shown in Fig. 4B, celecoxib alone markedly suppressed tumor progression, and this effect was further potentiated when combined with αPD-1. Kaplan–Meier analysis revealed that the combination regimen maintained a survival rate > 70% at day 60, significantly exceeding that of all other groups (Fig. 4C). MRI and ex vivo imaging demonstrated the smallest tumor volume (Fig. 4D and E) and lowest tumor weight (Fig. 4F) in mice receiving celecoxib plus αPD-1. Histological examination of stained tumor sections confirmed that the combination therapy most effectively induced cancer-cell death and inhibited proliferation (Fig. 4G).

### Celecoxib combined with αPD-1 activates a robust antitumor immune response after iRFA of HCC

**Fig. 5:**
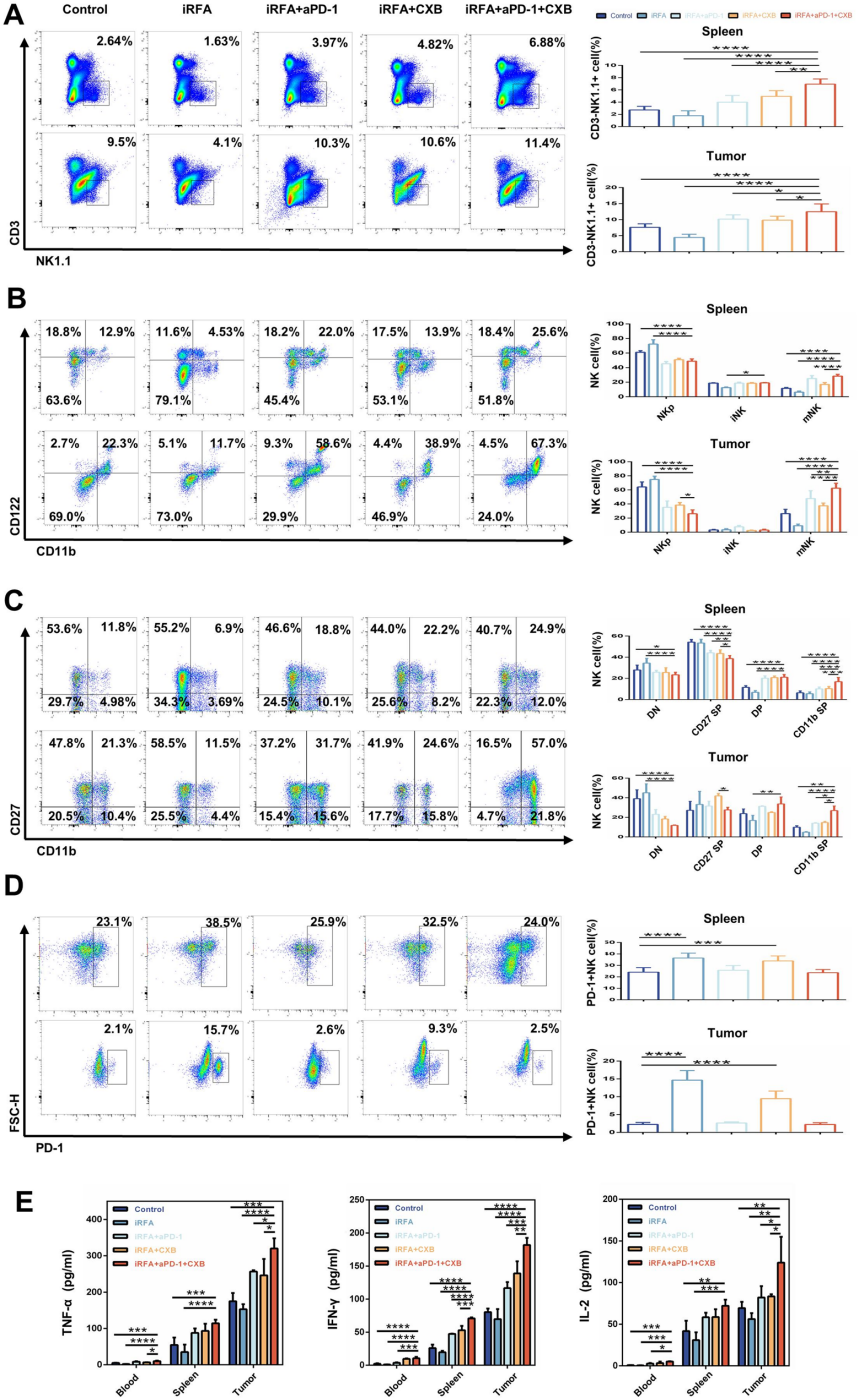
Immune Response Activation by Celecoxib and αPD-1 Combination Therapy. **A** Flow cytometric analysis of NK cell infiltration in spleen and residual tumor tissues. **B** Flow cytometric analysis of percentage of NKp, iNK, mNK in spleen and residual tumor tissues. **C** Flow cytometric analysis of percentage of four NK cell subsets in spleen and residual tumor tissues. **D** Flow cytometric analysis of PD-1 expression on NK cells derived from spleen (upper panel) and tumor (lower panel) tissues in different groups. **E** ELISA analysis of TNF-α, IFN-γ, and IL-2 production in blood, spleen, and tumor tissues. (*n* = 5, *p < 0.05; **p < 0.01; ***p < 0.001; ****p < 0.0001; iRFA, incomplete radiofrequency ablation; αPD-1, anti-PD-1 antibody; CXB, celecoxib; NKp, Natural Killer precursor; iNK, induced Natural Killer; mNK, mature Natural Killer)

Furthermore, the systemic antitumor immune response elicited by the combination regimen was evaluated. As depicted in Fig. 5A, flow cytometric analysis demonstrated significantly increased NK cell infiltration in residual tumors and spleen tissues in combination therapy group compared to the other four groups. Additionally, the percentage of mature NK (mNK) cells (CD11b+/CD122 + cells) in tumor and spleen tissues was significantly elevated in combination therapy group (Fig. 5B). Further analysis using classical NK cell typing suggested that celecoxib markedly increased the intratumoral frequency of the CD11b⁺/CD27⁻ NK subset, and this elevation was further amplified when αPD-1 was added (Fig. 5C). In addition, combined αPD-1 administration significantly reduced PD-1 expression on NK cells (Fig. 5D). Consistently, the combination regimen substantially elevated the concentrations of TNF-α, IFN-γ, and IL-2 in peripheral blood, spleen, and tumor tissue (Fig. 5E). Collectively, these results indicate that the combination of celecoxib with αPD-1 significantly enhances the antitumor immune response following iRFA for HCC.

### Abscopal-like effect and rechallenge test against tumors by the combined therapy of celecoxib with αPD-1

**Fig. 6:**
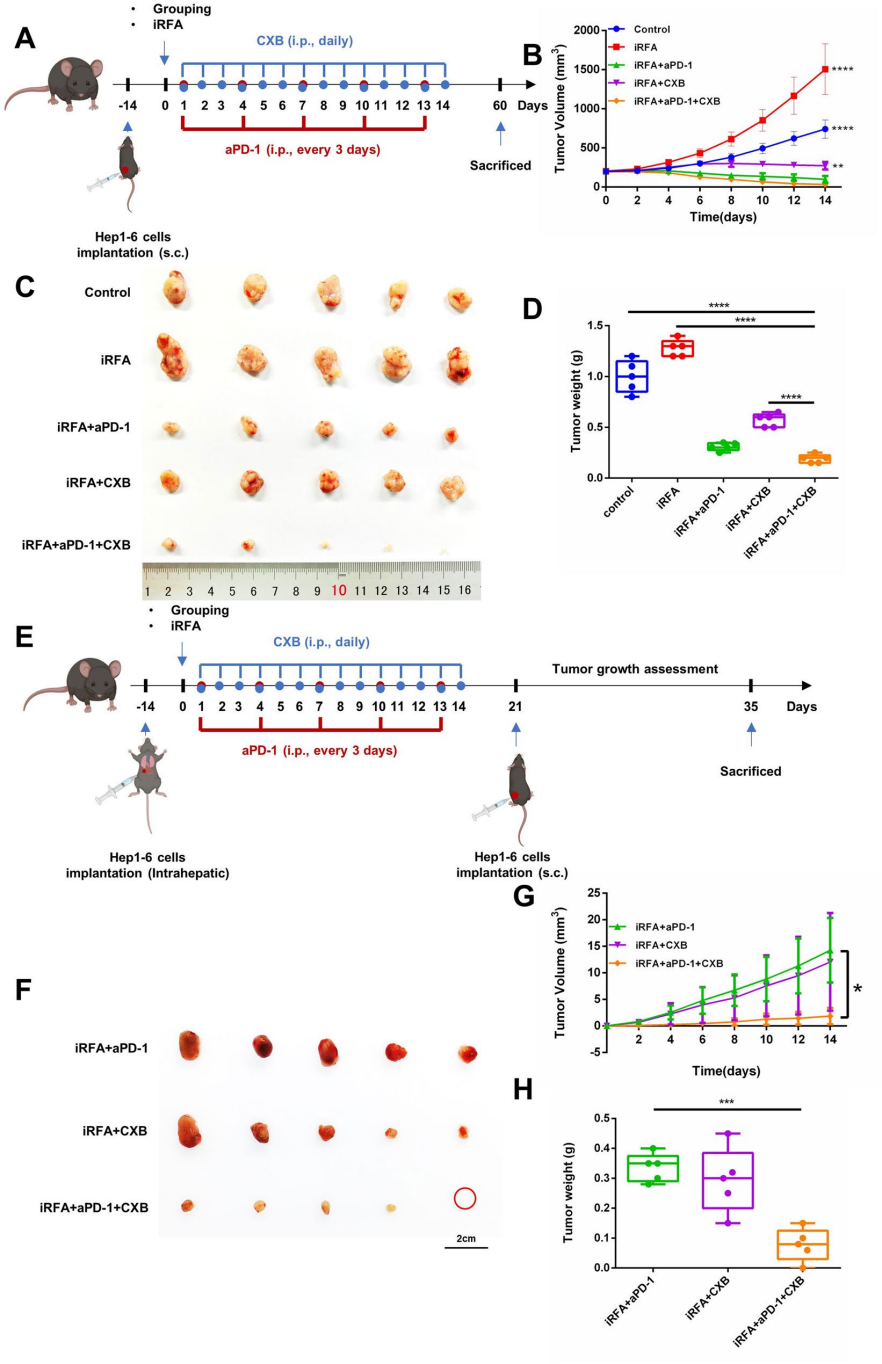
Abscopal-like Effect and Rechallenge Test of Combined Therapy of celecoxib and αPD-1 antibody. **A** Experimental design for evaluating the abscopal-like effect of combined therapy. **B** Tumor growth on the right dorsal flank of mice in different groups. **C** Images of harvested tumors from the abscopal-like effect experiment. **D** Tumor weights of abscopal tumors in different groups. **E** Experimental design for the rechallenge test. **F** Images of harvested tumors from the rechallenge test. **G** Changes of tumor volume on the left dorsal flank of mice in different groups. **H** Weights of tumors on the left dorsal flank of mice in different groups. (*n* = 5, *p < 0.05; **p < 0.01; ***p < 0.001; ****p < 0.0001; iRFA, incomplete radiofrequency ablation; αPD-1, anti-PD-1 antibody; CXB, celecoxib)

As depicted in Fig. 6A, a bilateral distant-tumor model was established to evaluate the abscopal-like effect. Tumors were harvested and weighed on day 14. As illustrated in Fig. 6B-D, celecoxib markedly suppressed growth of the non-treated distant tumors, which were significantly smaller than those in both the control and iRFA groups. Flow cytometric analysis suggested increased NK cell infiltration in the distant tumors of celecoxib-treated group (Supplementary Figure S1 A and B), with significantly higher proportions of mNK cells (CD11b + CD122+) and the CD11b + CD27- subset (Supplementary Figure S1C-F). Additionally, multiplex immunostaining further confirmed that celecoxib substantially inhibited tumor-cell proliferation and increased apoptotic indices relative to control and iRFA tumors (Supplementary Figure S2). These effects were further amplified when celecoxib was combined with αPD-1.

As described in Fig. 6E, a rechallenge test was conducted to assess whether the combination of celecoxib and αPD-1 antibody could activation antitumor immune memory. Notably, there were insufficient surviving mice in the control and single iRFA groups to proceed with the rechallenge test. Fourteen days after rechallenge, five mice from each of the remaining three groups (iRFA + αPD-1, iRFA + CXB, and iRFA + αPD-1 + CXB) were selected to monitor the growth of rechallenge tumors. The findings indicated that combination therapy (iRFA + αPD-1 + CXB) exhibited the most potent inhibitory effect on rechallenge tumor growth when compared to the other two groups (Fig. 6F-H).

### Evaluation of in vivo biosafety

**Fig. 7:**
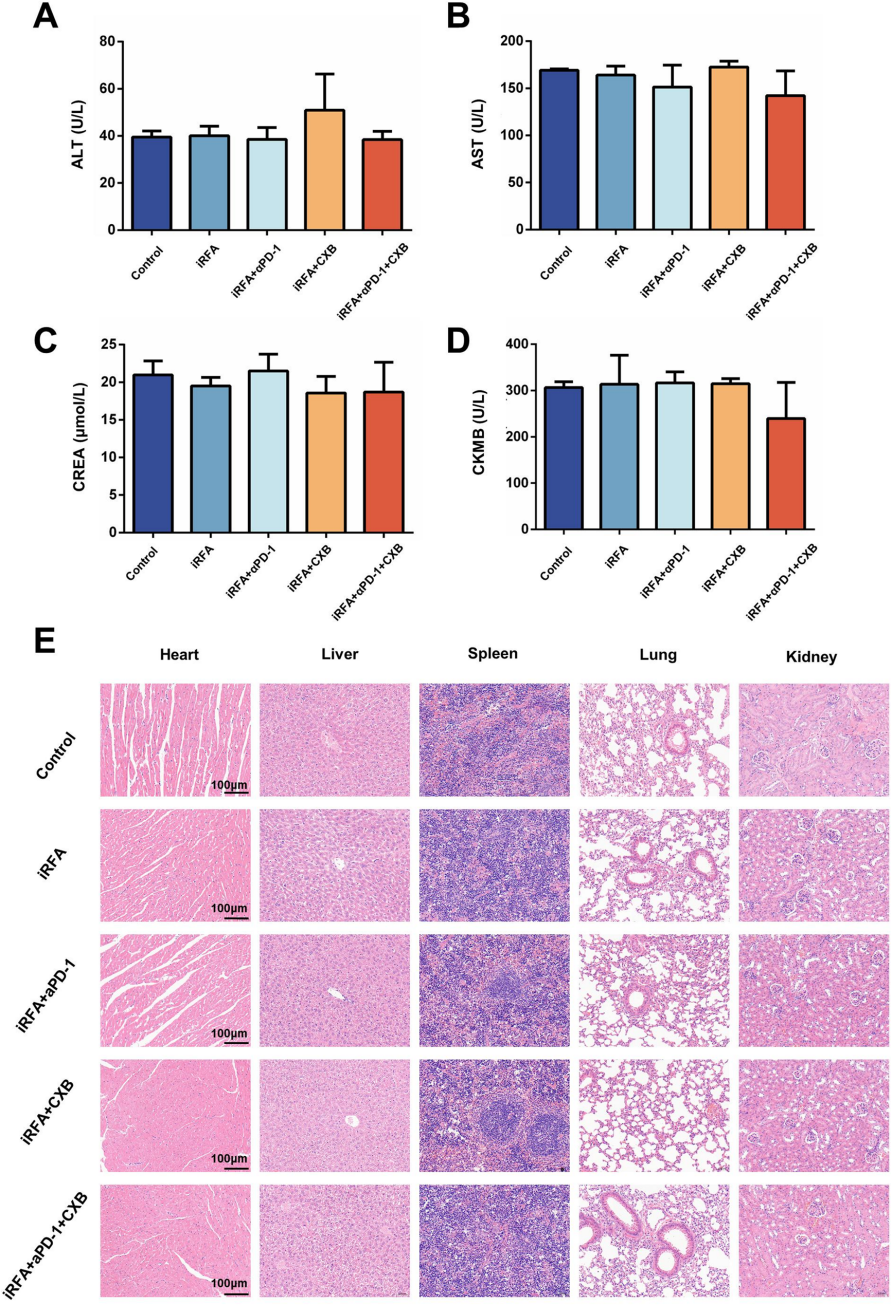
Safety Evaluation. **A-D** Biochemical analysis of liver function (ALT, AST), kidney function (CREA), and heart function (CKMB) in different treatment groups. **E** HE staining of major organs (heart, liver, spleen, lungs, kidneys) in mouse models from different groups. (*n* = 5, *p < 0.05; **p < 0.01; ***p < 0.001; ****p < 0.0001; ALT, alanine aminotransferase; AST, aspartate aminotransferase; CREA, creatinine; CKMB, creatine kinase MB; iRFA, incomplete radiofrequency ablation; αPD-1, anti-PD-1 antibody; CXB, celecoxib)

Assessments of liver function, kidney function, and heart function were conducted for each treatment group (Fig. 7A-D). The results demonstrated that the treatments had negligible effects on the liver (ALT, AST), kidneys (CREA), and heart (CKMB) of the mice. Histological examination via H&E staining revealed normal tissue morphology in major organs, including the heart, liver, spleen, lungs, and kidneys, with no apparent pathological alterations (Fig. 7E). Collectively, these findings indicate that the combination of celecoxib and αPD-1 antibody is well-tolerated and safe for treating residual cancer following iRFA for HCC.

## Discussion

RFA has become one of the primary treatment options for patients with unresectable liver cancer, with its minimally invasive nature, minimal damage to healthy tissues, short recovery time, repeatability, and broad applicability [19, 20]. However, numerous clinical centers have been reporting an increasing number of cases of HCC progression following RFA [21–23]. This phenomenon is associated with alterations in the tumor pathological microenvironment after incomplete RFA [24, 25]. In the treatment of HCC, incomplete ablation is a common issue that may lead to tumor recurrence and metastasis. To address this problem, researchers have been exploring more effective therapeutic strategies.

Celecoxib, a selective COX-2 inhibitor and nonsteroidal anti-inflammatory drug (NSAID), is widely used for the treatment of pain and inflammation [26]. Increasing evidence has revealed its potential antitumor effects. As a selective COX-2 inhibitor, celecoxib exerts antitumor effects by inhibiting the COX-2/PGE_2_ signaling pathway [27–29]. By reducing COX-2 expression in tumor tissues, celecoxib inhibits tumor angiogenesis and metastasis, thereby creating a more favorable tumor immune microenvironment for chemotherapeutic agents [30]. Prostaglandins generated by COX receptors have been proven to play a central role in cancer-related tissue inflammation [31]. Tissue inflammation is associated with several pathways known to promote tumor growth, which are upregulated following RFA [32, 33]. Thus, targeted inhibition of prostaglandin secretion to control tissue inflammation also represents a potential therapeutic target.

This study investigates the effects of combining celecoxib with αPD-1 on residual liver cancer after RFA through in vitro and in vivo experiments. First, in both experimental animals and human HCC tissues, we demonstrated that COX-2 expression in tumor cells is significantly upregulated following RFA, leading to increased PGE_2_ secretion, which is consistent with previous studies [8]. Furthermore, results show that celecoxib significantly inhibits COX-2 expression and downstream PGE_2_ secretion in tumor cells following RFA. αPD-1 block the binding of PD-1 on tumor-infiltrating NK cells and elevated PD-L1 on residual tumor cells, thereby synergistically enhancing the activity of NK cells and their cytotoxicity against tumor cells, ultimately prolonging the survival of tumor-bearing mice.

NK cells represent an essential element of the host’s innate immune system and are pivotal in the initial defense mechanisms against cancer [34, 35]. The intrinsic capacity of NK cells to swiftly identify and eradicate tumor cells in the absence of prior sensitization is of critical importance. NK cells exert cytotoxic effects through four main mechanisms: secreting cytokines to directly act on target cells; tumor-specific antibodies binding to CD16 on NK cells to induce ADCC effects [36]; releasing perforin and granzyme B to lyse tumor cells; and activating FASL, TRAIL, and their ligands to mediate death receptor pathways, thereby inducing target cell death. Elevated PGE_2_ following RFA reduces the expression of activating receptors NKp30 and NKp46 on NK cells, increases the expression of inhibitory receptor NKD1, and decreases surface protrusions on NK cells as observed under scanning electron microscopy. This leads to functional limitations and promotes tumor immune evasion. By inhibiting COX-2 activity, celecoxib effectively lowers PGE_2_ levels and restores the antitumor function of NK cells. Specifically, NK cells differentiate into mNK cells (CD11b+/CD122 + cells) and the CD11b+/CD27- subset with stronger antitumor capabilities. They express more cytolytic effector molecules such as TRAIL, FASL, Perforin, and Granzyme B, and secrete more antitumor immune factors like TNF-α and IFN-γ, thereby enhancing the body’s ability to kill tumors.

Recently, crosstalk between the PD-1/PD-L1 and COX-2/PGE₂ axes has been identified [37, 38]. Numerous studies have explored combining COX-2 inhibitors with immune checkpoint inhibitors. In murine melanoma models, pharmacologic COX inhibition with aspirin or celecoxib markedly potentiated the antitumor activity of αPD-1 antibody, indicating that targeting the COX-2/PGE₂ pathway may serve as an effective adjunct to ICI therapy [37]. Similarly, Naoya Maekawa et al. demonstrated in a canine melanoma model that dual blockade of the PD-1/PD-L1 and COX-2/PGE₂ pathways augments IL-2 and IFN-γ production by stimulated peripheral blood mononuclear cells, identifying this strategy as a promising means to enhance antitumor immunity in dogs [39]. Liu et al. combined rosmarinic acid–mediated COX-2 inhibition with ginsenoside-mediated interference of PD-1/PD-L1 engagement and achieved robust suppression of colorectal cancer metastasis in an MC38 pulmonary-metastasis mouse model [40]. However, to date, no study has evaluated the combination of a COX-2 inhibitor and immune checkpoint inhibitors in HCC. Gaurav Kumar et al. combined the COX-2 inhibitor celecoxib with RFA for liver cancer treatment. They found that the addition of celecoxib could reduce local activation of COX-2, lower the expression of RFA-related tissue inflammatory and angiogenic factors, and inhibit macrophage infiltration around the ablation area, thereby improving the efficacy of RFA [8]. However, the therapeutic effects of the combination of celecoxib and RFA are still not sufficiently pronounced and the safety of the combination therapy was not validated. Moreover, they primarily focused on the infiltration of inflammatory cells without delving into the activation status and functional changes of immune cells. Our study focuses on the elevated PD-1 and PD-L1 expression following RFA and further introduces αPD-1 to construct a combination therapy regimen. The results show that combination therapy regimen is superior to the combination of RFA and celecoxib or αPD-1 alone. To our knowledge, no prior studies have explored this specific combination therapy regiment in HCC, particularly for eradicating residual tumors after iRFA.

In this study, the safety of the combination therapy regimen was thoroughly evaluated. The results showed that the combination of RFA, celecoxib, and αPD-1 had no significant impact on liver, kidney, or heart function in mice, and no obvious pathological changes were observed in the morphology of major organs. This indicates that the combination of celecoxib and αPD-1 antibodies for treating residual tumors after RFA in HCC is safe and reliable.

While this study has yielded valuable findings, it may have certain limitations. First, although we verified the changes in COX-2, PGE_2_, and NK cell numbers in human HCC tissues after iRFA, no drug-efficacy validation has been conducted in humans. In addition, murine NK biology differs from that of humans. Therefore, it remains to be determined whether human NK cells respond in the same way. Second, this research primarily focuses on short-term efficacy assessments. Long-term outcome and safety data are still limited, yet such information is essential for clinical translation. Third, although celecoxib is a selective COX-2 inhibitor, off-target effects are possible. The optimal dose for humans must be established in prospective clinical trials.

In summary, the combination therapy of celecoxib and αPD-1 has demonstrated efficacy and safety in the treatment of residual tumors following RFA in HCC. This innovative therapeutic strategy holds promise for reducing the risk of tumor recurrence and metastasis after RFA in medium-to-large and irregular HCCs. Future research will further explore the clinical application value of this therapeutic regimen to provide stronger support for the treatment of liver cancer.

## Supplementary Information

Supplementary Material 1.

## Data Availability

No datasets were generated or analysed during the current study.
