# Supplementary material for: COX-2/PGE2 axis blockade with celecoxib enhances anti-PD-1 efficacy by activating natural killer cells for residual hepatocellular carcinoma after radiofrequency ablation
Source: J Exp Clin Cancer Res. 2025 Dec 17;44:321. doi: 10.1186/s13046-025-03582-6 (PMC12709788; doi:10.1186/s13046-025-03582-6)
Supplement: Supplementary file 1 — Supplementary Material 1. [file 13046_2025_3582_MOESM1_ESM.docx]

Contents

[Supplementary Appendix 1 Colony formation assay and scratch wound healing assay 2](#_Toc212135782)

[Supplementary Figure S1. Immune response activation of abscopal tumors. 3](#_Toc212135783)

[Supplementary Figure S2. Abscopal Effect of Celecoxib and αPD-1 Combination Therapy in a Mouse Model of HCC Tumors on the Right Dorsal Flank. 4](#_Toc212135784)

[Supplementary Table S1. Real‐time quantitative PCR primer sequences used in this study. 5](#_Toc212135785)

# Supplementary Appendix 1 Colony formation assay and scratch wound healing assay

A colony formation assay was conducted to evaluate the proliferative potential and colony-forming ability of cells. After co-culture, Hepa1-6 cell suspensions from the three groups were adjusted to a density of 500 cells/mL and seeded into 6-well plates. The cells were incubated for 2 weeks to allow colony formation. Throughout this period, the culture medium was replaced every 3 days, and the cell condition was regularly monitored. At the end of the incubation, cells were fixed with paraformaldehyde and stained with crystal violet solution to visualize the colonies. Colonies were then counted, and images of each well were captured and analyzed using ImageJ software.

The scratch wound healing assay was employed to assess cell migratory capacity. Hepa1-6 cells from the three co-culture groups were seeded at a density of 20,000 cells/mL in 6-well plates. Once the cells reached 90-100% confluence, a consistent wound was created by scratching the cell monolayer with a standard 200μL pipette tip. The detached cells were removed by gently washing with PBS. Subsequently, the plates were incubated in serum-free medium. Images of the wound width were taken using an inverted microscope at 0 and 48 hours for further analysis.


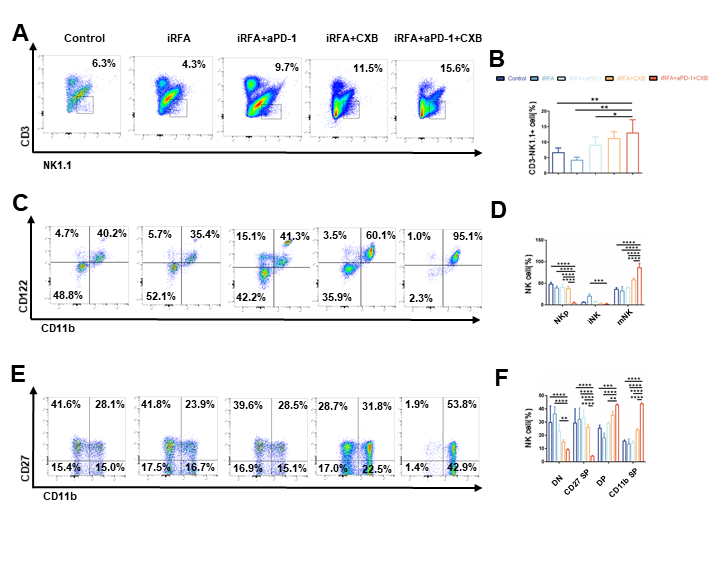


Supplementary Figure S1. Immune response activation of abscopal tumors. (**A, B**) Flow cytometric analysis of NK cell infiltration in abscopal tumor tissues. (**C, D**) Flow cytometric analysis of percentage of NKp, iNK, mNK in abscopal tumor tissues. (**E, F**) Flow cytometric analysis of percentage of four NK cell subsets in abscopal tumor tissues. (n=5, *p<0.05; **p<0.01; ***p<0.001; ****p<0.0001; iRFA, incomplete radiofrequency ablation; αPD-1, anti-PD-1 antibody; CXB, celecoxib; NKp, Natural Killer precursor; iNK, induced Natural Killer; mNK, mature Natural Killer).

Supplementary
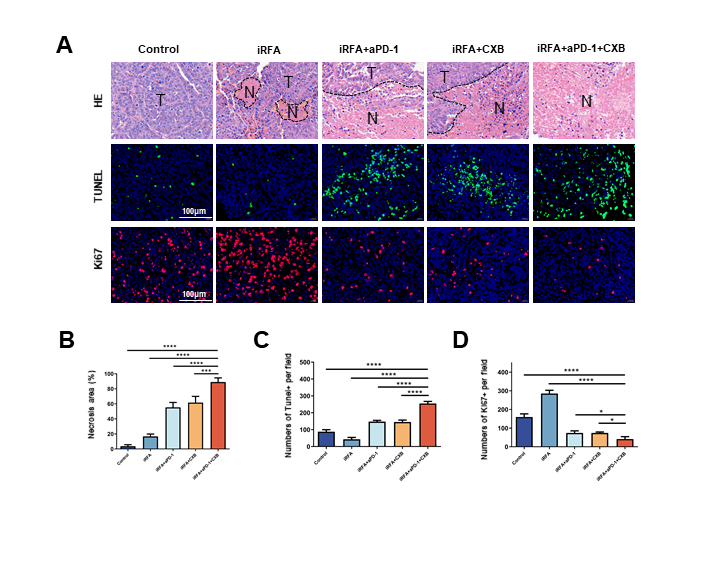
Figure S2. Abscopal Effect of Celecoxib and αPD-1 Combination Therapy in a Mouse Model of HCC Tumors on the Right Dorsal Flank. (**A-D**) HE, TUNEL and Ki-67 staining analysis of tumors. (n=5, *p<0.05; **p<0.01; ***p<0.001; ****p<0.0001; iRFA, incomplete radiofrequency ablation; αPD-1, anti-PD-1 antibody; CXB, celecoxib).

# Supplementary Table S1. Real‐time quantitative PCR primer sequences used in this study.

| Gene name | Forward primer sequence 5’ to 3’ | Reverse primer sequence 5’ to 3’ |
| --- | --- | --- |
| TRAIL | TGGCTGTAACTTACGTGTACTT | TCATACTCTCTTCGTCATTGGG |
| FasL | CACAGCATCATCTTTGGAGAAG | GTACAGCCCAGTTTCATTGATC |
| Perforin | GCTATCGTTAGTGCTAGTGGAT | ATCTGTCTGATGCGTATCCAAT |
| Granzyme B | GAAAGTGCGAATCTGACTTACG | TTGTTTCGTCCATAGGAGACAA |
| GAPDH | CCTCGTCCCGTAGACAAAATG | TGAGGTCAATGAAGGGGTCGT |

*TRAIL, tumor necrosis factor-related qpoptosis-inducing ligand; FASL, Fas ligand; GAPDH, glyceraldehyde-3-phosphate dehydrogenase.*
